# Nanoparticle Metrology of Silicates Using Time-Resolved Multiplexed Dye Fluorescence Anisotropy, Small Angle X-ray Scattering, and Molecular Dynamics Simulations

**DOI:** 10.3390/ma17071686

**Published:** 2024-04-07

**Authors:** Daniel Doveiko, Alan R. G. Martin, Vladislav Vyshemirsky, Simon Stebbing, Karina Kubiak-Ossowska, Olaf Rolinski, David J. S. Birch, Yu Chen

**Affiliations:** 1 Photophysics Group, Department of Physics, University of Strathclyde, Glasgow G4 0NG, UKdjs.birch@strath.ac.uk (D.J.S.B.); 2 EPSRC Future Continuous Manufacturing and Advanced Crystallisation National Facility, University of Strathclyde, 99 George Street, Glasgow G1 1RD, UK; alan.r.martin@strath.ac.uk; 3 School of Mathematics and Statistics, University of Glasgow, Glasgow G12 8QQ, UK; vladislav.vyshemirsky@glasgow.ac.uk; 4 PQ Silicas UK Limited, Warrington WA5 1AB, UK; 5 ARCHIE-WeSt, Department of Physics, University of Strathclyde, Glasgow G4 0NG, UK

**Keywords:** sodium silicates, fluorescence anisotropy decay, particle metrology, rhodamine 6G, rhodamine B, SAXS, MD, adsorption, molecular dynamics

## Abstract

We investigate the nanometrology of sub-nanometre particle sizes in industrially manufactured sodium silicate liquors at high pH using time-resolved fluorescence anisotropy. Rather than the previous approach of using a single dye label, we investigate and quantify the advantages and limitations of multiplexing two fluorescent dye labels. Rotational times of the non-binding rhodamine B and adsorbing rhodamine 6G dyes are used to independently determine the medium microviscosity and the silicate particle radius, respectively. The anisotropy measurements were performed on the range of samples prepared by diluting the stock solution of silicate to concentrations ranging between 0.2 M and 2 M of NaOH and on the stock solution at different temperatures. Additionally, it was shown that the particle size can also be measured using a single excitation wavelength when both dyes are present in the sample. The recovered average particle size has an upper limit of 7.0 ± 1.2 Å. The obtained results were further verified using small-angle X-ray scattering, with the recovered particle size equal to 6.50 ± 0.08 Å. To disclose the impact of the dye label on the measured complex size, we further investigated the adsorption state of rhodamine 6G on silica nanoparticles using molecular dynamics simulations, which showed that the size contribution is strongly impacted by the size of the nanoparticle of interest. In the case of the higher radius of curvature (less curved) of larger particles, the size contribution of the dye label is below 10%, while in the case of smaller and more curved particles, the contribution increases significantly, which also suggests that the particles of interest might not be perfectly spherical.

## 1. Introduction

Sodium silicates are very versatile inorganic chemicals, manufactured on industrial scale by combining silica sand and soda ash (sodium carbonate) under high temperatures. They are often used in coating and bonding applications when in an aqueous solution. Additionally, they exhibit various attractive characteristics, such as being odourless, non-toxic, having high strength and rigidity, being resistant to high temperatures, and having an overall low cost [1]. Furthermore, they serve as a primary source for producing colloidal silica and silica gels, and are widely employed commercially as thickeners and absorbents. Given their versatility, there is an urgent need to deepen understanding of their chemistry to enhance control over production processes, optimize them, and thereby improve overall sustainability [2]. 

Important characteristics of silicates are the ratio of silica to soda and the size of the dissolved species. The gold standard used by industrial manufacturers to identify the presence of different oligomer species in sodium silicates is ^29^Si nuclear magnetic resonance (NMR) spectroscopy [3]. However, this method does not provide any quantitative data regarding the size of different species. Furthermore, there are forty-eight different silica structure species identified up to date, corresponding to only 85% of silicon in the solution [2]. Recently, there have been some promising developments in determining the size and shape of different oligomer species using small-angle X-ray scattering (SAXS) and dynamic light scattering (DLS), where it was shown that the dominating fraction of silica is present in small amorphous clusters with a nominal radius of 0.7 nm and a minor component consisting of larger colloidal species of around 15 nm in radius [4,5,6]. Unfortunately, both of these methods are far from ideal and have significant drawbacks: DLS becomes difficult when trying to determine the particle sizes below the 10 nm range [7], and SAXS is expensive and complex [8]. Given small particles within sodium silicates, measurements must be performed at the nanoscale with nanometre precision, commonly referred to as nanometrology [9]. Furthermore, characterizing sodium silicates presents an additional layer of complexity compared to typical nanoparticles like colloidal silica or gold nanoparticles. This complexity arises from the variety of structures and their polydispersity, which in turn limits the precision of nanometrology [2].

The alternative approach discussed here and developed originally in our laboratory is based on the measurement of time-resolved fluorescence anisotropy of fluorescent probes, which utilizes the relationship between the silica particle size and the rate of the probe’s rotational diffusion when bound to the silica particle [10]. This fluorescence technique offers high sensitivity and has an appropriate timescale (ps to ns), and, due to the high variety of fluorescent probes available, the experiment can be adapted to the specification of the medium to be researched, for example, pH and temperature. Furthermore, the required fluorescence time-resolved instrumentation is relatively inexpensive and easy to use.

However, previous anisotropy studies have shown [11] that the variety of different mechanisms of depolarization, such as dye diffusion on the particle surface and wobbling, may cause additional complications and require careful data interpretation and analysis before the particle size can be determined.

Here we exploit the fact that rhodamine 6G (R6G) physically binds to silica particles [11,12], while rhodamine B (RB) does not bind due to the electrostatic repulsion from its carboxylic acid group [13]. This allows using the rotational time of the non-binding RB to independently determine the microviscosity, which can then be used with the rotational time of R6G to estimate the size of the particle, as previously applied by D. Tleugabulova et al. [14] using R6G (excitation 495 nm) and pyranine (excitation 404 nm) to study Sol-to-Gel transitions in sodium silicate. In our case both R6G and RB can also be excited with a single light source at around 500 nm if required. The effect of this multiplexed dye approach is to eliminate the decrease in abundance of the free probe with time and the concomitant increase in the error in the measured viscosity and measured particle radius, as occurred in previous studies using a single dye probe [15]. To ensure a more precise determination of the silica particle size, we used two methods of modifying microviscosity in the samples: dilution of the original sample in water, and change of temperature. Furthermore, we focus on raw, poly-dispersed sodium silicates and validate the anisotropy-measured particle size against SAXS measurements combined with Molecular Dynamics (MD) simulations to investigate the impact of the dye’s size on the measured average size of the silica nanoparticles. The findings further support the interpretation of time-resolved fluorescence anisotropy in terms of silica particle size and provide insight as to its measurement limits in this application. Moreover, the methods described herein can be applied to other nanoscale systems, e.g., the G4PAMAM complex with the anti-cancer drug 5-fluorouracil, which change their conformation in different conditions. By enabling the identification of such alterations without the need for complex SAXS measurements [16], this approach presents the pharmaceutical industry with a powerful tool for precise characterization of nanoscale structures, facilitating enhanced quality control and optimization of drug formulations to bolster efficacy and safety.

## 2. Materials and Methods

Sample Preparation. The sodium silicate solution selected for the investigation was purchased from Sigma-Aldrich (Darmstadt, Germany) as a product of Millipore (Darmstadt, Germany). According to the product description, the solution in its undiluted form has a pH of 11–11.5 at 50 g/L at 20 °C and dynamic viscosity of 130 mPa∙s. It contains 25.5–28.5% SiO_2_ and 7.5–8.5% Na_2_O (3.7 silica to soda ratio), and has a density of 1.296–1.396 g/mL at room temperature. The content of Fe and other heavy metals (such as Pb) is ≤50 ppm [17].

R6G (Bioreagent, Suitable for Fluorescence), Sodium Silicate Extra Pure (Millipore), and NaOH 1N volumetric standard solution were purchased from Sigma-Aldrich, and RB (99%, Laser Grade) was purchased from Fisher Scientific (Waltham, MA, USA). Both fluorescent probes were added directly to the stock solutions (100%—not diluted) of the sodium silicate samples, creating samples of the dye concentration of 10 µM.

The diluted sodium silicate samples were prepared by mixing them with distilled water starting from nine-parts stock silicate/NaOH and one-part water (90%) until nine-parts water and one-part stock silicate solution (10%) has been reached. The dye was added after the dilution at 10 µM concentration.

Samples in plastic cuvettes, 1 × 1 × 4 cm, with relevant solutions were prepared immediately before the measurements. The sodium silicate solution is quite stable in the sealed container, but the continuous exposure to CO_2_ present in the atmosphere has a tendency of lowering the pH because of formation of carbonic acid in the silicate. When combined with sample evaporation, this leads to gelation. Therefore, the cuvettes were covered with parafilm to minimize the exposure of the samples to CO_2_ to prevent gel formation.

Temperature-Controlled Measurements. The samples were prepared by taking 3 mL of stock sodium silicate and adding 10 µM of the probe directly into the sample without prior dilution. The temperature in the chamber was controlled using NesLab RTE-111 Bath circulator connected to the sample holder. The sample temperature was monitored using an Omega HH804 (Omega Engineering, Norwalk, CT, USA) probe, which was kept in the cuvette at all times during the experiment. The sample chamber was purged at low temperature with nitrogen to prevent water condensation.

Time-resolved measurements. Fluorescence intensity decay measurements to obtain both the lifetime and anisotropy data were based on the time-correlated single-photon counting (TSCPC) [18,19] technique, and performed using a HORIBA-IBH (Glasgow, UK) DeltaFlex system with both excitation and emission Seya-Namioka monochromators which incorporate holographic diffraction gratings in order to minimise the detection of scattered light. The R6G-labelled samples were excited using a HORIBA-IBH NanoLED [20] with a centre wavelength of 494 nm, pulse duration of 1.5 ns and the repetition rate of 1 MHz. The emission was collected at 548 nm. RB samples were excited using a NanoLED laser diode with peak wavelength of 509 nm, pulse duration of 200 ps, and repetition rate of 1 MHz, with the fluorescence being collected at 570 nm. Accurate R6G and RB fluorescence lifetime measurements have been previously successfully demonstrated using LED excitation [21]. Fluorescence decay data were collected using the FluoroHub-A electronics containing a time-to-amplitude (TAC) converter operated in reverse mode with the start-to-stop rate kept below 1% to minimize data pile-up [18,19]. The individual fluorescence decays were measured at a magic angle 54.7° in order to eliminate orientational effects [18,19].

Time-resolved decay data analysis. Various methods exist for analysing time-resolved decays, each offering unique advantages. For instance, the maximum entropy method excels in representing decay without imposing a specific model [22]. Alternatively, the lifetime distribution approach simplifies decay analysis by reducing required parameters [23], while the maximum likelihood model effectively handles data noise through Poisson statistics [24]. In this study, we employ a multi-exponential least-squares model known for its simplicity and robustness, with demonstrated exceptional performance over time [25,26,27]. Furthermore, for fluorescent dyes exhibiting fluorescence intensity decays following a simple mono-exponential model, each recovered component corresponds to a fluorophore exposed to a different environment [28]. However, in the case of protein, the justification of employing the methods presented herein becomes more challenging due to the inherently complex photophysics involved [29]. Strictly speaking, *n*-exponential decay times describes *n* excited states, but of course, even if this is not appropriate to the kinetics, multi-exponentials can still be used to describe a decay. In fact, for the purpose of calculating anisotropy decay, the model used does not have to describe the actual kinetics, but merely provide a good fit to the data, as demonstrated by the χ^2^ goodness of fit. This is a real strength of the fluorescence approach to nanometrology we are using. 

Reconvolution software (v1) generally assumes *n*-exponential decay times [18,19], but can be modified to also include the detection of scattered excitation light [30,31] when fitting the fluorescence decay parameters to the raw decay data, according to the formula:

(1) $$F\left(t\right)=a+bL\left(t+∆\right)+c\int_{0}^{t}L\left(t+∆-t^{\prime }\right)I\left(t^{\prime }\right)dt^{\prime }.$$

where *L*(*t*) is the instrumental response function (IRF), *a* is the background noise count level, *b* is the contribution of the scattered light, and *c* is the scaling parameter. The temporal difference in response between the IRF and the fluorescence decay caused by the effect of their wavelength differences on the light detector and light path can be approximated using a linear shift parameter Δ, while the *I*(*t*)

(2) $$I\left(t\right)=\sum_{i}^{n}b_{i}\left(t\right)\exp\left[-\frac{t}{t_{i}}\right]$$

is the *n*-exponential model of the decay.

As time-resolved fluorescence anisotropy theory has been widely reviewed, we present only a short summary here that is of relevance to nanoparticle rotation.

The anisotropy decay function *R*(*t*) can be generated by recording vertically *I_V_*(*t*) and horizontally *I_H_*(*t*) polarized fluorescence decay curves. Both decays are then combined, forming the anisotropy function [32]:

(3) $$R\left(t\right)=\frac{I_{V}\left(t\right)-I_{H}(t)}{I_{V}\left(t\right)+2I_{H}(t)}$$

The sum and the difference of vertically and horizontally polarized fluorescence decay curves have to be reconvoluted and fitted to obtain the anisotropy decay curve. First, the denominator, which describes the fluorescence decay, is fitted using the nonlinear least square method, and the resultant impulse response is iteratively convoluted with *R*(*t*) to provide the best fit to the numerator using appropriate models for rotation until the minimum in χ^2^ is determined. The anisotropy *R*(*t*) was calculated using the HORIBA IBH DAS6 (v 6.8) software package. The polarizer dwell time was set to 60 s, and the photons were counted until the peak difference of 10,000 counts was reached. For both sets of excitation and emission wavelengths, the G-factor correction for the relative detection efficiencies of *I_V_*(*t*) and *I_H_*(*t*) was applied.

In the simplest case of spherical rigid rotor in an isotropic medium, the fluorescence depolarization due to the Brownian rotation can be described by

(4) $$R\left(t\right)=R_{0}\exp\left(-\frac{t}{\emptyset_{r}}\right).$$

For one photon excitation, the initial anisotropy $R_{0}$ has a value of 0.4, and the rotational correlation time ∅*_r_* can be described using the Stokes–Einstein equation

(5) $$\emptyset_{r}=\frac{\eta V}{kT}$$

where *T* is the temperature, *k* is the Boltzmann constant, *η* is the microviscosity of the medium, and *V* is the hydrodynamic volume. Assuming spherical shape of the rotating particles, we can express the hydrodynamic radius *R_p_* as

(6) $$R_{p}=\sqrt[{3}]{\frac{3kT\emptyset_{r}}{4\pi \eta }}.$$

If the radius of the spherical rotor *R_p_* is known, the rotational time can be used to determine the microviscosity of the medium using

(7) $$\eta =\frac{3kT\emptyset_{r}}{4\pi R_{p}^{3}}.$$

In the approach here, we use the rotational correlation time of RB to calculate the microviscosity of the medium from Equation (7) and then use it in Equation (6) to estimate the radius of R6G–silicate particles.

Small-angle X-ray scattering. The SAXS data were collected with a Xenocs Xeuss 2.0 SAXS/WAXS (Grenoble, France) using Cu Kα^1^ radiation Genix3D microfocus source with a wavelength of 1.5406 Å, with an operating voltage of 50 kV and operating current of 0.6 mA. Single-reflection multilayer optics with 2D collimation were used, and the scattered beam was collimated through two sets of motorised scatterless slits with variable apertures. A DECTRIS Pilatus 1M detector (Baden, Switzerland) was used to collect the SAXS data with the sample to detector distance calibrated at 2507.35 mm and a pixel size of 172 µm. Five 30 min images were recorded and accumulated by measuring the scattering intensity as a function of scattering vector q = 2πsinθ/λ, where 2θ is the scattering angle and λ is the wavelength of the scattering beam. Samples in their original concentration were prepared in stainless steel sample holders sealed with Kapton dots to limit the air exposure. The sample-detector path was *in-vacuo* to avoid any scattering or absorption from air. The scattering curves of the samples were corrected for the background scattering from the instrument, Kapton dots, and residual atmosphere [33]. The data were analysed using BioXTAS RAW (v2.2.1) [34] software.

MD Protocol. Following the MD simulations and protocol presented previously [12], the starting structure for the R6G was taken from the protein databank entry 2v3l.pdb [35], which was modified to match the experimental one. Furthermore, the partial charges were corrected to match the values obtained by Chuichay et al. [36]. The SNPs were built using the CHARMM-GUI Nanomaterial Modeller [37,38], while the rest of the system preparation was performed using VMD [39]. All simulations were performed using NAMD3 CUDA version [40,41]. Interface Force-Field (FF) [42] was used for the SNPs and CHARMM36 [43] was used for the rest of the system components. As usually done in MD simulations, the system minimization was a two-step process. Initially, a water only minimization was performed, consisting from 1000 minimization steps followed by 100 ps of equilibration in 300 K temperature. Afterwards, the whole system underwent a minimization, consisting of 10,000 minimization steps followed by 30 ps of heating to 300 K and 270 ps of thermalisation with a 1 fs step. For the production runs, the integration step was set to 1 fs to minimize errors and ensure system stability, with the total length of the trajectory being 100 ns. VdW cut-off was set to 12 Å, while the Particle Mesh Ewald (PME) was used to calculate the electrostatic interactions. The system was solvated using the TIP3P [44] water model, while the internal water molecule vibrations were constrained. The neutralization was done using NaCl, resulting in six Cl^−^ ions added to neutralize the cationic charge of R6G, while all the Na^+^ ions present in the system came from ionizing the SNP to the appropriate pH via deprotonating Si-O-H. The anisotropic cell fluctuations ensured that the desired pressure of 1 atm at 300 K was reached.

## 3. Results and Discussion

### 3.1. Steady-State Measurements

**Figure 1:**
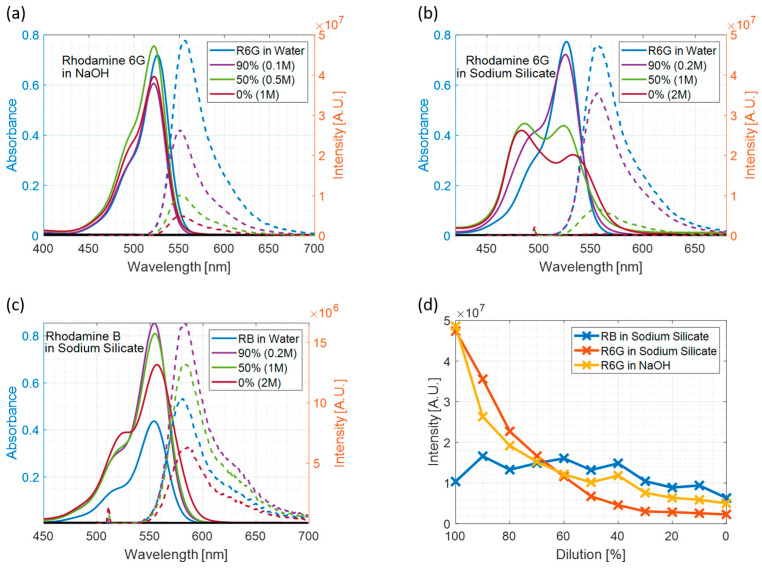
Steady-State results where percentages represent the level of dilution and the molarity represents the concentration of NaOH in the sample. The solid line represents the absorption spectra while the dashed line represents emission spectra. (**a**) R6G absorption and emission spectra in water at various concentrations of NaOH; (**b**) R6G absorption and emission spectra in sodium silicate; (**c**) RB absorption and emission spectra in sodium silicate; (**d**) RB emission intensity as a function of dilution. Samples in water were prepared by diluting stock solution in distilled water to 10 µM dye concentration.

The results of steady-state measurements of R6G samples in water at various concentrations of NaOH and sodium silicate and RB samples in sodium silicate are shown in Figure 1.

In the case of R6G in sodium silicate (Figure 1b), there is the emergence of a new peak at around 470 nm in the absorption spectrum, which is growing with increasing concentration of sodium silicate and pH in the sample. This effect is widely known [45,46], as R6G tends to form non-fluorescent aggregates (most probably dimers), also known as H-type. Interactions of the dye molecules with the polar surface of the silica structures is very clearly represented in the emergence of the higher energy peak in the absorption spectra. R6G dimers have a strong absorption band at a shorter wavelength when compared with monomers. However, they do not fluoresce, and are mainly responsible for quenching the fluorescence. This process does not occur in at this concentration where there are no silica particles present (Figure 1a), hence the R6G aggregation due to high dye concentration can be excluded. Both sets of plots show the dominating absorption peak at 530 nm, typical for R6G, and a vibronic shoulder at around 500 nm, which overlaps with the H-Type dimer absorption band.

Samples were excited at 494 nm for R6G and 509 nm for RB to measure the emission spectra. Fluorescence emission spectra were recorded in 1 nm increments. The acquired spectra show that the emission intensity is heavily dependent on the sodium hydroxide content in the sample. Namely, with increasing concentration of NaOH, the intensity of the R6G emission decreases, which suggests that increasing pH with the addition of NaOH quenches the R6G fluorescence. On the other hand, when looking at the absorption and emission spectra of RB in sodium silicate, the trend of decreased intensity of both the absorption and emission spectra with pH is much less evident, as further visualised in Figure 1d. It seems to be evident that in cases when R6G is used, the emission intensity drops when the sample is less diluted, which corresponds to increasing the concentration of NaOH. The drop in R6G intensity in the basic regime is caused by the quenching of R6G excited states due to the high concentration of OH^−^ ions, which happens in a multi-step way. Initially, at pH between 10 and 12, T_1_ triplet state is affected, and when pH rises above 12, the S_1_ singlet state is additionally depopulated through a non-radiative decay channel [47].

Based on the steady-state measurements, it might be concluded that R6G forms non-fluorescent aggregates when exposed to silica particles, but the dominating factor controlling its fluorescence intensity is the concentration of sodium hydroxide in the sample.

### 3.2. Fluorescence Decays

**Figure 2:**
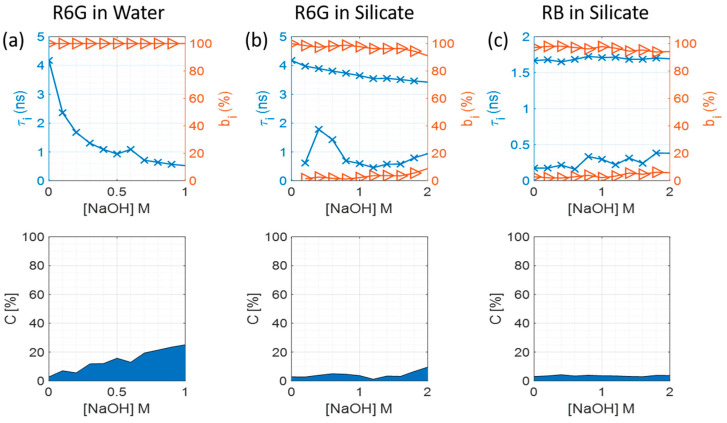
Fluorescence decay times (τ_i_, marked by blue crosses), their contributions (b_i_, marked by orange triangles), and scattered excitation light contribution (c) depending on the NaOH concentration for each sample group, where the blue area represents the scatter while the white area represents the fluorescence. (**a**) R6G in water at different concentrations of NaOH; (**b**) R6G in sodium silicate; (**c**) RB in sodium silicate.

Fluorescence lifetime measurements were performed and fitted to several exponential decay models. The 1-exponential model (R6G in water) and 2-exponential model (R6G in silicate and RB in silicate) were found to be the simplest functions that result in acceptable values for the best fit of χ^2^ < 1.2 (see Tables S1 and S2 in Supplementary Information). The changes in fluorescence decay components and their contributions when the concentration of NaOH and sodium silicate is changing are shown in Figure 2.

The pure NaOH samples show the biggest decrease in R6G lifetime at higher pH (see Figure 2a). In the case of R6G in sodium silicate samples, the τ_1_ component is also shortening as the sodium silicate concentration increases, which corresponds to the increasing amount of NaOH in the sample that is present in the manufactured sodium silicates (see Figure 2b). It is worth noting that when silicate particles are present in the system, the drop in τ_1_ component is not as significant as for pure NaOH samples, which suggests that the presence of silica particles in the samples helps to maintain the decay time of R6G. On the other hand, RB lifetime stays constant in all the samples, and is only slightly affected by the presence of sodium hydroxide or silica particles. Additionally, the scattering increases with the growing concentration of NaOH, which suggests an increase in nanoscale particulates. It is also important to discuss the emergence of the second lifetime component in the sodium silicate samples. As is illustrated on Figure 2b, in the presence of sodium silicates, there is a second lifetime component with a fluorescence decay time shorter than 1 ns. It might be that this component corresponds to free dye in the sample, as its decay component at 1 M of NaOH closely matches the value of R6G in NaOH at 1 M concentration. The slight increase of its contribution at the 2 M concentration might also suggest that most of the dye has adsorbed to the silica particle surface; therefore, any additional dye that would be added to the sample would not be adsorbed, but will stay in the sample in its free form instead. Furthermore, RB also exhibits a biexponential behaviour, suggesting that there is an additional excited state present. The origin of this second component is not entirely clear; nevertheless, it may be that a fraction of RB could adsorb on some of the silicate oligomers. Given this short component is a small fraction of the total emission, its presence does not seem to impact our particle size measurements, as we will show in the following sections.

### 3.3. Time-Resolved Fluorescence Anisotropy in Diluted Samples

**Figure 3:**
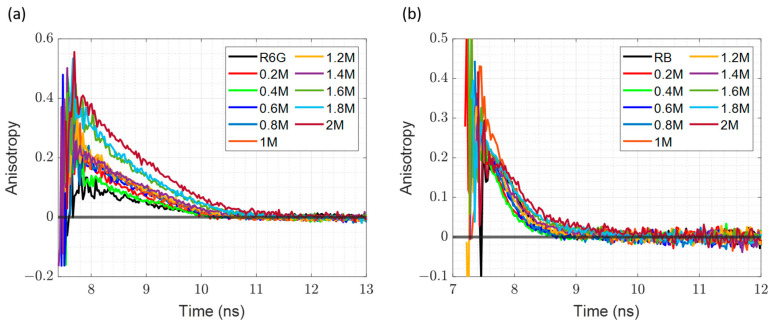
Time-Resolved Fluorescence Anisotropy Decays as a function of sodium hydroxide concentration. (**a**) R6G–silicate complex; (**b**) RB in sodium silicate samples.

The time-resolved fluorescence anisotropy decays of R6G–silicate complexes and RB in silicate, the latter being used to determine the microviscosity of the samples, are presented on Figure 3. Both dyes are comparable in size, and the only difference comes from the presence of the negatively charged carboxylic acid group in RB, which will result in electrostatic repulsion and hence will not bind to the negatively charged silicate particle [13]. The obtained plots are consistent with this, indicating that the decay of anisotropy is notably longer in the R6G–silicate complex when compared with the RB decays. Furthermore, it is important to discuss the discrepancy of the r_0_ values from the theoretical value of 0.4. Theoretically, in time-resolved anisotropy measurements, the r_0_ value starts at 0.4. In our case, as indicated by Figure 3, in the case of R6G, this is correct only for the 2 M, 1.8 M, and 1.6 M samples, while for the case of RB, the initial anisotropy in all cases is lower than 0.4. Those lower values are well-known to be due to rapid intramolecular reorientation from the absorption to the emission dipole.

The results of anisotropy decay fittings to (4) are shown in Tables S3 and S4. Given that the χ^2^ values are acceptable in most cases, we deemed that mono-exponential model of anisotropy decay is appropriate. The measurements for the pairs of samples of the same presence of silicate were performed: one for R6G (attached to silicates), and the second for RB (freely rotating in the solution). By combining those two measurements, and assuming that the microviscosity in each pair is the same, we can determine the particle size in the sodium silicate solutions using the method described previously [13]. The results are listed in Table 1.

To precisely determine the hydrodynamic radii of both R6G and RB, we prepared two aqueous dye solutions of 10 µM concentration each and performed time-resolved anisotropy measurements. The data were collected until specified count difference was obtained, and the measurements were repeated in 5000 count difference steps until we acquired six decays, starting with 5000 counts and continuing all the way until 30,000 were reached. The data were fitted next, the corresponding hydrodynamic radii obtained and the average size of the dye were calculated. Resulting anisotropy measurements of both dyes in water gave the hydrodynamic radius of 5.9 ± 0.6 Å for R6G and 5.7 ± 0.2 Å for RB (assuming viscosity of water at 20 °C 1 cP and the temperature 293.15 K), which agree well with values obtained previously using the picosecond polarization grating technique and multi-colour dual-focus fluorescence correlation spectroscopy experiments [48,49]. As both dyes are comparable in size, slower decays of the R6G samples suggest that R6G is indeed attached to silica particles (Figure 3). Having the radius of the RB, we were able to determine the microviscosity of each sample. Then, using the obtained microviscosities and the rotational time of particles labelled with R6G allowed us to calculate the size of the labelled silica particles.

Based on calculated microviscosities using RB rotational times and the estimated particle sizes using the R6G rotational times, as listed in Table 1, it is evident that the measured sizes are significantly smaller in the samples diluted to 1.6 M NaOH and lower, when compared with the undiluted and 1.8 M samples. Additionally, the recovered sizes are the upper limit, as our measured size of R6G is 5.9 ± 0.6 Å and we do not know how the particle orientates on the silica surface.

**Figure 4:**
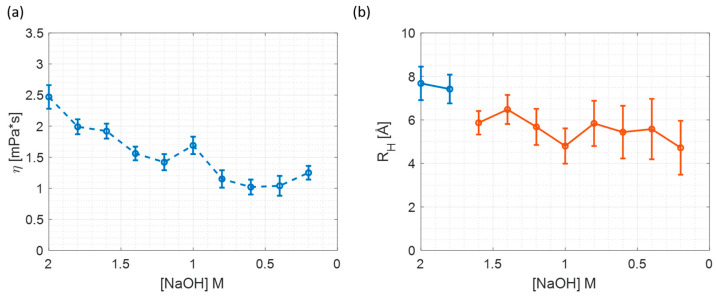
The microviscosity and particle size dependence on sample dilution. (**a**) Microviscosity; (**b**) particle size.

The microviscosity and particle size changes upon sample dilution are illustrated in Figure 4. The microviscosity is going down upon dilution, which is expected, as is the detected particle size. Moreover, starting from 1.6 [NaOH] M, the particle size is equal to the free R6G size. It might be explained by the fact that the dilution decreases the amount of silicate particles in the sample, while the dye concentration is maintained at the value of 10 µM; therefore, the amount of free dye is growing. Hence, the anisotropy decay will be dominated by the free dye.

The formation of the non-fluorescent H-type dimers seems to not affect the particle size measurements in any way. When looking at the UV-Vis absorption spectra in Supplementary Figure S1, we can clearly see that the H-type dimers are present in all samples from 2 M to 0.6 M of NaOH, but neither the anisotropy decays shown in Figure 3 nor the particle sizes listed in Table 1 show any discrepancies. This proves that only R6G monomers fluoresce when measuring the particle sizes, and the dimers have no visible effect on the course of the experiment.

### 3.4. Fluorescence Intensity Decays at Different Temperatures

**Figure 5:**
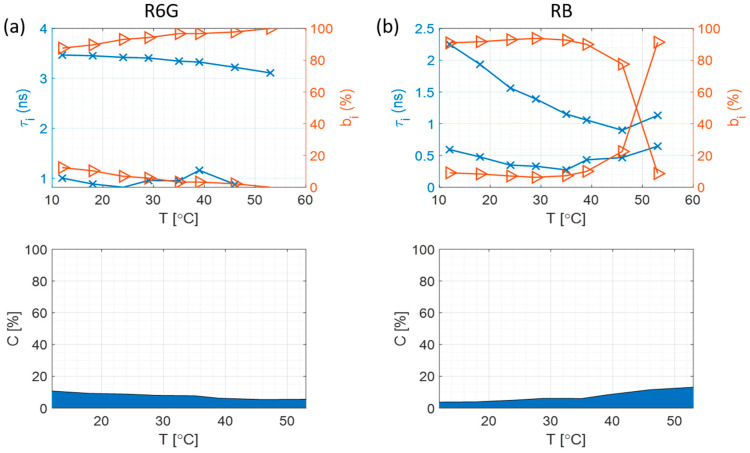
Fluorescence decay components (τ_i_, marked by blue crosses), their contributions (b_i_, marked by orange triangles), and scattered excitation light contribution (c) depending on the temperature for each sample group at pH 11, where the blue area represents the scatter and the white area represents the fluorescence. (**a**) R6G in sodium silicate; (**b**) RB in sodium silicate.

As in the case of dilution experiments fluorescence, decay measurements were performed and fitted to several models. The 2-exponential model was found to be the simplest that can generate acceptable values of the χ^2^ (Tables S5 and S6). Figure 5 shows the changes in fluorescence decay components and their relative contributions when the temperature is changed. It is noted that both dyes behave in a very different way. R6G (Figure 5a) decay times stay almost the same over the whole range of temperatures, suggesting that the complexation with silica particles protects it from the effects of collisional quenching at high temperatures. Moreover, the scatter goes down at higher temperatures.

In the case of RB molecules (Figure 5b), the most abundant decay time drops sharply with the increase in temperature as a result of faster molecular movement, more frequent collisions, and hence stronger dynamic quenching. Furthermore, the amount of scattered light increases with increasing temperature, which is a result of the short decay time at high temperatures, suggesting that at higher temperatures the anisotropy decay measurement may not be so accurate.

Lastly, as it was the case in Section 3.2 with diluted sodium silicate samples, here there are also two decay components present, which suggests that at all times there is a fraction of the dye which is free (shorter component), while the adsorbed dye decay component dominates in the sample (longer component).

### 3.5. Time Resolved Fluorescence Anisotropy at Different Temperatures

**Figure 6:**
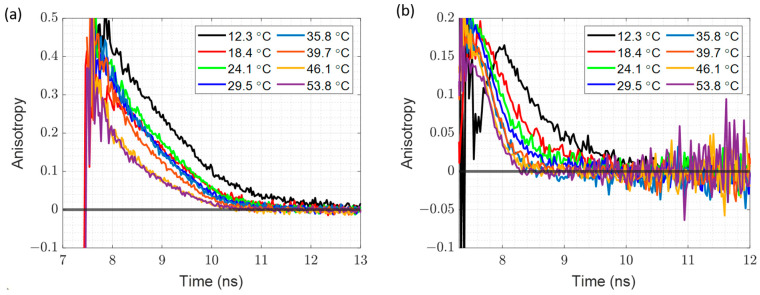
Time resolved fluorescence anisotropy decay at different temperatures of (**a**) R6G–Sodium Silicate Complex; (**b**) RB in Sodium Silicate solution.

The fluorescence anisotropy decay of R6G–silicate complexes and RB in silicate at different temperatures are shown in Figure 6. In both cases, the decay follows a very clear trend: at lower temperatures the decay is longer, while at higher temperatures the decay is faster. This is directly correlated with the microviscosity, which is higher at low temperatures and lower at high temperatures. As a result, here we have a much more controlled experiment, where the only factors that are changing are the temperature and viscosity, with sample composition staying the same during the whole duration of the experiment.

Supplementary Tables S7 and S8 provide the details of the fitting results for both samples at different temperatures. As it was in the case of dilution, given that the χ^2^ values are acceptable, we deemed that the mono-exponential model was again appropriate. As in the case of the dilution experiment, the rotational time of RB reported on the microviscosity at different temperatures. and the rotation time of the R6G–silicate complex combined with microviscosity, allowed the determination of the size of the complex.

Table 2 lists the calculated microviscosities using RB rotational time and the particle sizes determined using R6G rotational times. It is reassuring to note that the size is more-or-less constant over a wide range of temperatures when compared with the dilution experiment.

**Figure 7:**
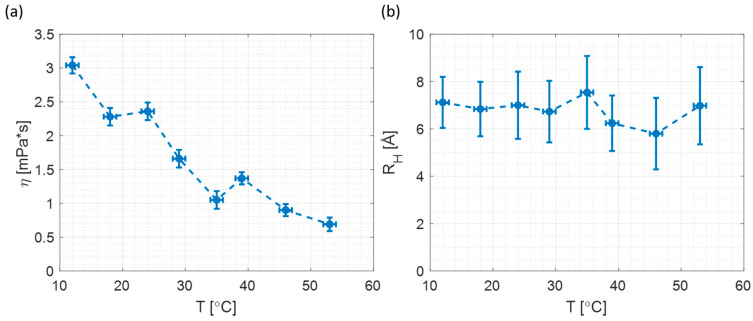
The microviscosity and particle size dependence on temperature (**a**) Microviscosity; (**b**) Particle Size.

The temperature dependence of microviscosity and particle radius is shown in Figure 7. The microviscosity (Figure 7a) drops with increasing temperature. Comparison with Figure 4a indicates that microviscosity at 2 M of NaOH matches the microviscosities at 18 °C and 24 °C (Figure 7a).

Furthermore, a comparison of Figure 4b and Figure 7b suggests that in the case of changing temperature, the detected particle size is constant and the size remains constant at the wide range of the temperature. Additionally, the measured particle size at various temperatures matches the results obtained for 2 M and 1.8 M samples in the dilution experiment. All the above suggests that changing the temperature is a better method for changing viscosity when measuring silicate particle size, as the sample composition does not change with the temperature.

The subsequent step is to determine the size of the silica particle (without the R6G label dye). The determined hydrodynamic radius of R6G is 5.9 ± 0.6 Å, and the size of the silicate-R6G complex is 7.0 ± 1.2 Å. If it is assumed that the dye attaches to the surface of the particle without any alterations to its shape, then the silica particle size would be 1.1 Å, which is less than a water molecule size (~2.8 Å [50]). It is worth mentioning that the Si-O bond length is longer than that of O-H [51]; therefore, the simplest estimation leads to impossible results. This in turn strongly suggests that the dye must adjust its orientation and shape upon binding to the silica particles.

### 3.6. Multiplexed Time-Resolved Measurements

We prepared a sample of pure sodium silicate containing 10 µM of both R6G and RB to simplify the method of multiplexing two dyes even further. Using λ_E_x = 494 nm, we measured both fluorescence intensity decays and anisotropy decays, starting from λ_Em_ = 525 nm and measuring in 5 nm increments until λ_Em_ = 595 nm. The measured fluorescence intensity decays and time-resolved anisotropy decays are shown on Figures S6 and S7.

**Figure 8:**
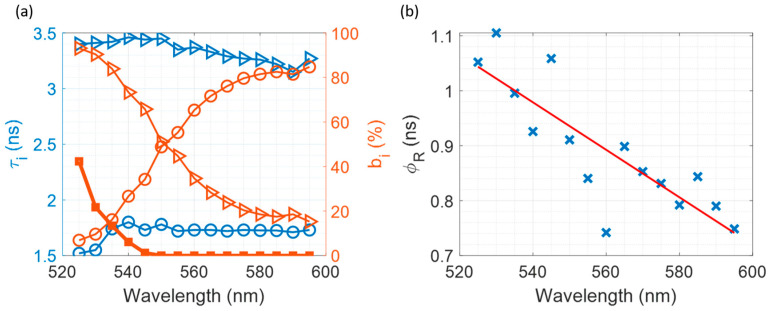
Fitting results for the Multiplexed Time-Resolved Measurements. (**a**) Fluorescence decay components (τ_i_) and their contributions (b_i_) as a function of λ_Em._, while the thick line with squares shows the contribution of the scattered excitation light. Triangles represent recovered R6G parameters, while circles represent the recovered RB parameters. (**b**) Recovered rotational times as a function of λ_Em_.

The results of fitting fluorescence intensity decays to a bi-exponential function and anisotropy decays to a mono-exponential function are shown in Figure 8.

The wavelength-dependent decay time component contribution is clearly seen in Figure 8a, where, at λ_Em_ = 525 nm, the R6G contribution to the overall decay is over 90% while RB is below 10%. The opposite is observed when the λ_Em_ is set to 595 nm, where the decay is dominated by the RB fluorescence with its contribution being over 80% and the R6G contribution dropping below 20%. Between those two points, the contribution of the R6G gradually decreases and RB increases as we move towards longer wavelengths, which is to be expected, as the emission wavelength at 595 nm is far from the peak emission of R6G at 548 nm, and vice versa for the shorter wavelengths.

The rotational times acquired from the anisotropy decay fitting to a mono-exponential model are shown in Figure 8b. The recovered rotational times have a clear wavelength dependence, and can be separated into three groups. The first group is for the emission between 525 and 545 nm, where the decays are dominated by the R6G, hence the recovered rotational times are dominated by the R6G–silicate complex. The second group is for the emission range of 580 nm to 595 nm, where our decay is dominated by the RB, which is freely rotating in the solution and reports on the microviscosity of the solution. Finally, the third group are the decays in between the first and second group, where we see comparable contributions from both dyes, and the recovered rotational time is the mixture of the two dyes, one adsorbed to the particle surface and the other free in the solution. This is caused by the simultaneous fluorescence of both dyes, resulting from a significant overlap between their absorption and emission spectra.

Upon closer analysis, we can see that the precision with which we can measure the rotational time of the R6G–silica complex is limited by the scattered excitation light (see Figure 8a). At shorter wavelengths, the contribution from the scattered light grows exponentially, and initial anisotropy is above the theoretical limit of 0.4 (see Figure S7) [52]. On the other hand, as the scattering drops to zero at longer wavelengths and initial anisotropy decreases below the theoretical limit of 0.4 (see Figure S7), the only factors limiting the precision of RB rotational time measurement are the excitation source power and repetition rate. As a result, it is possible to minimize the R6G contribution even further by moving to even longer wavelengths, and thereby obtaining a more precise measurement of microviscosity. Finally, using the average value from the second group of the rotational times (free RB in solution) and Equation (7), we were able to determine the microviscosity of the sample η = (3.0 ± 0.5) mPa∙s. While using the average value from the first group (R6G-silica complex) and Equation (6), we recovered the hydrodynamic radius of the particle R_H_ = (7.1 ± 0.9) Å. Both of these results agree with the obtained values for both the η and R_H_ discussed in Section 3.3 and Section 3.5. It is important to mention that although two measurements were needed to determine particle size successfully, due to the fact that both dyes can be excited using a single excitation wavelength, this measurement could also be performed using a T-format spectrometer were we can monitor two emission wavelengths simultaneously [53,54], or across the whole fluorescence spectrum using a linear SPAD array combined with a spectrograph [55,56]. The choice of binding and non-binding dyes with overlapping but not identical absorption and fluorescence spectra, as demonstrated here, offers a lot of flexibility in optimising precision when measuring viscosity and particle hyrodynamic radius.

### 3.7. Small Angle X-ray Scattering and Comparison with Time-Resolved Anisotropy Measurements

**Figure 9:**
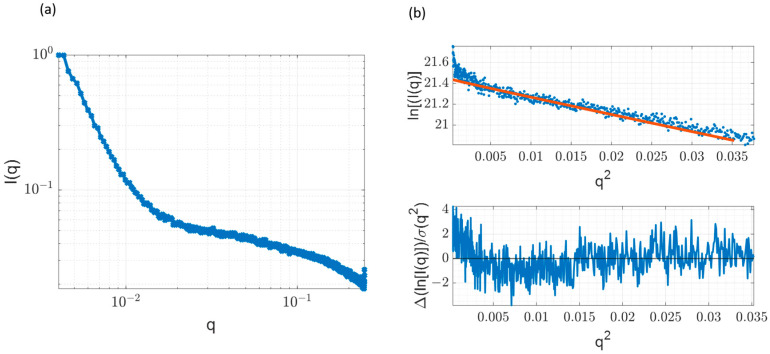
SAXS Results. (**a**) Corrected sample scattering profile. (**b**) Guinier fit of the sample with corresponding residuals plot. Blue dots represent the experimental data and the orange line represents the Guinier fit.

To cross-validate our anisotropy measurements and independently determine the average particle size of the sodium silicate, we performed SAXS measurements on the unlabelled stock solution. Figure 9 shows the summed and corrected SAXS data over five 30 min images. Using the reduced SAXS data, the R_g_ (radius of gyration) was calculated by fitting the corrected scattering profile of the undiluted silicate to the Guinier model:

(8) $$lnI\left(q\right)\approx -\left(\frac{R_{g}^{2}}{3}\right)q^{2}$$

where *I*(*q*) is the scattering intensity and *q* is the scattering vector. Due to the exponential behaviour of the Guinier approximation, the R_g_ values can be determined by plotting ln(I) vs. q^2^, which is shown in Figure 9b. The obtained value for the R_g_ was 6.50 ± 0.08 Å with R^2^ equal to 0.95. This agrees well with results obtained by J. Nordström et al. [4] for the 3.3 silica-to-soda-ratio silicate, exhibiting very close composition to the silicate user herein. Furthermore, a characteristic turn in the residuals plot is evident (Figure 9b) and caused by the polydispersity in the silicate species present in the sample [5].

As expected, the radius of gyration is lower than the hydrodynamic radius due to the inclusion of a hydration shell in the case of R_H_ (6.50 ± 0.08 Å vs. 7.0 ± 1.2 Å). However, it is important to note that the ratio R_g_/R_H_ is between 0.75–0.87, which is very close to the reference value of 0.77 obtained for the solid sphere [57]. Nevertheless, the obtained R_g_/R_H_ ratio is not centred around the 0.77 value, but slightly displaced towards 1.0, which might suggest a slightly elongated shape of the particles. The details regarding the shape of the complex are further investigated via the MD method and are described in the next section.

Lastly, the effect of the free dye in the sample when performing the anisotropy measurements needs to be considered as well. Due to the lack of strong intrinsic fluorescence and the need to label sodium silicates, the fraction of free dye will be present in the solution, contributing to the anisotropy decays. As a result, the measured average rotational time and resulting particle size will be smaller than that it would be in the case when there is no labelling dye present.

### 3.8. R6G Adsorption to Silica Nanoparticles

To fully understand the influence of the R6G size on the particle size determined using fluorescence anisotropy decay, we performed a range of MD simulations and measured the size of the complex to elucidate, on an atomistic level, the impact of the dye on the measured size.

**Figure 10:**
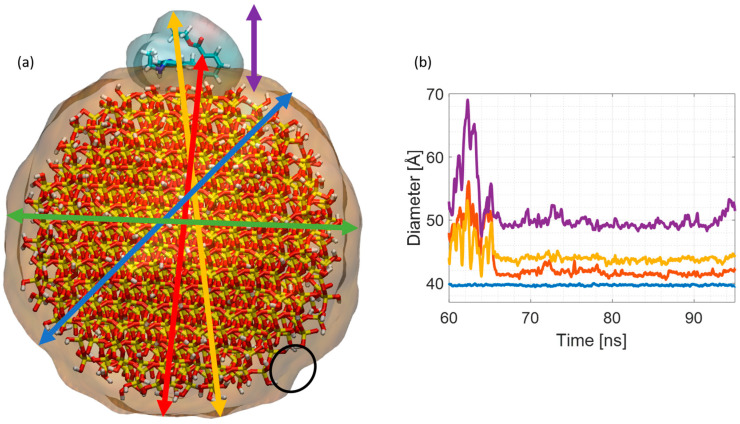
40 Å diameter SNP size measurements. (**a**) SNP–R6G complex. The black circle indicates local SNP surface irregularity, while the arrows indicate directions of distance measurements. The colour code: green and blue indicate the SNP diameter measured in two different directions, red indicates the distance between R6G xanthene core atom and the SNP atom on its opposite side, yellow points the largest distance between any R6G and SNP atom, while purple shows the maximal thickness of the adsorbed R6G molecules. All atoms are represented by thick sticks coloured by atom type: oxygen is red, silica is yellow, hydrogen is white, carbon is cyan, while nitrogen is blue. To indicate the size of the complex, the dye’s surface is shown with transparent representation. (**b**) Measured diameter of the SNP–R6G complex as a function of the simulation time. Colour code corresponds to the arrows code, while the purple line is simply a sum of the R6G thickness itself and the SNP diameter.

The measured diameter of the silica nanoparticle (SNP) alongside the diameter of the nanoparticle–R6G complex as a function of the simulation time is shown in Figure 10. In the case of the 40 Å diameter particle, even when the biggest possible distance between the furthest R6G and a silicate oxygen atom on the other side is taken into consideration, the dye contribution to the measured complex size is in the range of 10% (yellow line). However, by looking at the size of the R6G on its own and adding it to the SNP size, it is evident that the obtained value is significantly larger than the actual measured size of the R6G–SNP complex (purple line). This suggests that the shape of the SNP plays a crucial role when measuring the size. Moreover, the dye may not lie entirely flat on the surface, but marginally adjusts its shape to match that of the nanoparticle. The complex size is therefore smaller (yellow line) than the added sizes of separated counterparts (purple line). Similar analysis for 20 Å diameter SNPs is presented in Figure S6.

The situation is somewhat different in the case of smaller SNPs. The size contribution of R6G is significantly larger in these examples, and can be up to 50% of the measured size. The main reason is that 20 Å SNPs are significantly more curved when compared with 40 Å diameter SNPs. R6G, which is relatively rigid at its xanthene core plane, therefore cannot bend enough to match precisely 20 Å SNPs’ curvature. Furthermore, our recent studies have shown [12] it is impossible to have a layer of the R6G formed on the SNP surface because R6G adsorbs on SNP only via its xanthene core and because of the requirement for antiparallel orientation of the constituent molecules dipole moments. Additionally, as proved with the experimental results presented herein and confirmed with our previous MD work [12], the R6G dimers do not have any impact on the measured size, as they cannot interact with SNPs due to the aforementioned restrictions based on geometric constraints and dipole moment orientation. Finally, it is important to mention the shape of the SNP. In all cases, we considered that the SNP was perfectly spherical. However, this may not be the case. At 20 Å–40 Å scale, the atomistic structure and discrepancies on the atomic scale of the nanoparticle may also have some impact. One of such irregularities is indicated in Figure 9a by the black circle, and a small (~4 Å) discrepancy in diameter measured in different direction is illustrated in Figure 10b (blue and green lines). Based on that, we might anticipate that if the nanoparticle would be more ellipsoidal, then the dye contribution would be strongly impacted by the adsorption location. Namely, the dye size contribution to the size of entire complex would be smaller than measured here if R6G would adsorb on the less curved part of the ellipsoid, and probably similar to the contribution measured for spherical particles if the adsorption would occur on the more curved ellipsoid end. In a recent study conducted by G. Hu et al., it was shown using SAXS that for silicates with a ratio less than 4.2, the primary particles are ellipsoidal, which confirms our findings and further justifies why the dye impact on the measured size is small, compared to the MD-obtained results [6].

The discrepancy between the MD results and SAXS results allows us to draw a few important conclusions. The aforementioned simulation results demonstrate what is perhaps obvious, i.e., that the impact of R6G on the measured size is strongly influenced by the size of the labelled particle where the dyes contribution grows significantly with decreasing SNP size. This trend, however, is not observed when comparing the anisotropy and SAXS-obtained results, where R_g_ = 6.50 ± 0.08 Å (no R6G) while the R_H_ = 7.0 ± 1.2 Å (with adsorbed R6G), suggesting that the adsorbed dye has only a fractional contribution to the measured average particle size. Additionally, the obtained R_g_/R_H_ ratio is not centred around the 0.77 value, but slightly displaced towards 1.0, which is the case for elongated particles. This discrepancy in MD results comes from the fact that modelled particles used in the simulations were considered spherical, as modelling non-spherical and amorphous particles is very complex due to the lack of well-defined structures and parameter files, and therefore would require additional parametrization using quantum chemical methods. Therefore, the MD results presented here should be treated as an indicator of possible trends observed in the experiments. Due to the matching chemical composition and physical properties (charge, hydrophobicity, etc.) of crystalline SNPs used above, and the amorphous sodium silicates used in the experiments, the outcome of the simulations involving elongated SNPs made from amorphous silica would most probably result in a very comparable outcome to the one discussed above. Nonetheless, performing simulations involving elongated amorphous SNPs would most definitely help to broaden the understanding of this labelling mechanism. Considering all of the above, and the fact that R6G can only marginally adjust its shape in the case when the SNPs have smaller curvature, this suggests that the measured particles are elongated and R6G potentially adsorbs to the side of the SNP which is longer and has smaller curvature, allowing it to marginally adjust its orientation and marginally adjust its shape to match the curvature of the nanoparticle. In fluorescence anisotropy decay measurements, it is of course the hydrodynamic radius which determines the kinetics, and this effectively can take no account of a structure being non-spherical unless much higher statistical precision is obtained than that required for the metrology objective described here.

## 4. Conclusions

We have presented two approaches based on time-resolved fluorescence anisotropy that allow the determination of particle size of sodium silicate oligomers. The viscosity has been altered in two ways: by sample dilution, and by changing the temperature of the sample, the latter offering advantages. Finally, we showed that the particle size can be successfully measured using a single excitation wavelength when both dyes are present in the sample simultaneously. The measured particle size for Rhodamine 6G agrees with previous work [58], which leads to confidence that the measured sizes are not greater than reported here. Moreover, the data imply that the dye can marginally adjust its shape upon binding. We performed SAXS measurements on the undiluted sample to cross-validate the anisotropy results reported here. Guinier analysis of the SAXS data allowed for determination of the radius of gyration, which is equal to 6.50 ± 0.08 Å. This result agrees well with the previous results obtained for sodium silicates, and it simultaneously confirms the results and conclusions obtained from the anisotropy measurements. Finally, to get a better insight into the mechanism of sodium silicate particle labelling, we performed MD simulations of 40 Å and 20 Å diameter nanoparticles. MD trajectories showed that R6G can marginally adjust its shape to match the curvature of the nanoparticle. This implies that in the case of bigger and less curved particles the impact of the dye on the measured complex size is up to 10%, while for the more curved smaller particles, that size contribution grows substantially and can potentially be up to 50%. Combining all of the results above, we can speculate that there is a high possibility that the particles of interest are not perfectly spherical because of the agreement between anisotropy and SAXS measurements and the shape adjustment observed in MD simulations. This is consistent with a previous study [6].

In summary, provided the pH and dye are compatible, i.e., the dye is stable at highly alkaline pH and maintains a sufficiently long lifetime in that environment, the simple methods described here allow efficient determination of average silicate oligomer particle sizes. Furthermore, as already pointed out, a simple multi-exponential model can be utilized to describe the fluorescence decay kinetics, as the use of fluorescence anisotropy decay does not require an understanding of complex kinetics; the fluorescence is just providing a marker in time, related to particle rotation. A major benefit is that the samples are relatively simple to label and do not require any modifications that may alter the oligomer speciation, thus providing a simple method of monitoring silicates. Furthermore, the methods presented herein can be adapted to other systems involving labelled nanoparticles to determine the average size in a more simple and cost-effective manner. It is important to note that due to the complexity of sodium silicates and the variety of structures present, the method is limited to measuring the average size and cannot distinguish between different species within the silicates or determine size distributions.

## Figures and Tables

**Table 1:** Calculated microviscosities using RB rotational time, and the estimated upper size limit for the particle sizes using R6G rotational time.

SiO_2_ (%) (Estimated)	[NaOH] M (Estimated)	Rotational Time of Non-Binding Probe (RB) (ns)	Microviscosity (mPa∙s)	Rotational Time of Binding Probe (R6G) (ns)	Silica Particle Size (Å)
27.0	2.0	0.54 ± 0.04	2.47 ± 0.19	1.16 ± 0.03	7.7 ± 0.8
24.3	1.8	0.46 ± 0.03	1.99 ± 0.12	0.84 ± 0.02	7.4 ± 0.7
21.6	1.6	0.41 ± 0.03	1.92 ± 0.12	0.40 ± 0.01	5.9 ± 0.5
18.9	1.4	0.35 ± 0.02	1.56 ± 0.11	0.44 ± 0.02	6.5 ± 0.7
16.2	1.2	0.32 ± 0.03	1.42 ± 0.13	0.27 ± 0.01	5.7 ± 0.8
13.5	1.0	0.40 ± 0.03	1.69 ± 0.14	0.19 ± 0.02	4.8 ± 0.8
10.8	0.8	0.30 ± 0.03	1.15 ± 0.14	0.24 ± 0.01	5.8 ± 1.0
8.1	0.6	0.25 ± 0.03	1.02 ± 0.12	0.17 ± 0.02	5.4 ± 1.2
5.4	0.4	0.26 ± 0.02	1.04 ± 0.16	0.19 ± 0.003	5.6 ± 1.4
2.7	0.2	0.22 ± 0.04	1.25 ± 0.11	0.14 ± 0.02	4.7 ± 1.2

**Table 2:** Calculated microviscosities using RB rotational time, and the estimated upper limit of the particle sizes using R6G rotational time.

T (°C)	Rotational Time of Non-Binding Probe (RB) (ns)	Microviscosity (mPa∙s)	Rotational Time of Binding Probe (R6G) (ns)	Silica Particle Size (Å)
12 ± 1	0.72 ± 0.03	3.04 ± 0.12	1.17 ± 0.03	7.1 ± 1.1
18 ± 1	0.53 ± 0.03	2.28 ± 0.13	0.76 ± 0.03	6.8 ± 1.1
24 ± 1	0.54 ± 0.03	2.36 ± 0.13	0.82 ± 0.06	7.0 ± 1.4
29 ± 1	0.37 ± 0.03	1.66 ± 0.13	0.51 ± 0.03	6.7 ± 1.3
35 ± 1	0.23 ± 0.03	1.05 ± 0.13	0.44 ± 0.03	7.5 ± 1.5
39 ± 1	0.29 ± 0.02	1.37 ± 0.09	0.32 ± 0.03	6.2 ± 1.2
46 ± 1	0.19 ± 0.02	0.90 ± 0.09	0.18 ± 0.03	5.8 ± 1.5
53 ± 1	0.14 ± 0.02	0.69 ± 0.10	0.22 ± 0.03	7.0 ± 1.6

## Data Availability

Data are contained within the article and Supplementary Materials. Any additional data needed will be shared on a reasonable request to the corresponding author.

## Supplementary Materials

The supporting information can be downloaded at: https://www.mdpi.com/article/10.3390/ma17071686/s1, Figure S1: UV-Vis absorption Spectra of (a) R6G at different concentrations of NaOH; (b) R6G in Sodium silicate containing different concentrations of NaOH; Figure S2: Emission Spectra (λex = 494 nm) of (a) R6G at different concentrations of NaOH; (b) R6G in Sodium silicate containing different concentrations of NaOH; Figure S3: Excitation Spectra (λem = 590 nm) of (a) R6G at different concentrations of NaOH; (b) R6G in Sodium silicate containing different concentrations of NaOH; Figure S4: Fluorescence intensity decays of (a) R6G in NaOH, (b) R6G-silicate complex; (c) RB in sodium silicate; Figure S5: Fluorescence intensity decays at different temperatures (a) R6G-Silicate complex; (b) RB in Sodium Silicate; Figure S6: Fluorescence intensity decays for the multiplexed measurements; Figure S7: Anisotropy decays for the multiplexed measurements; Figure S8: 20 Å diameter SNP measurement results. Colour code follows the one introduced on Figure 9 while the sum of separated sizes is ignored for clarity; Table S1: R6G Parameters recovered from Fluorescence Intensity Decay fitting; Table S2: RhB Parameters recovered from Fluorescence Intensity Decay Fitting; Table S3: Anisotropy Fitting Results for R6G-Silicate complex; Table S4: Anisotropy Fitting Results for RhB in Sodium Silicates; Table S5: R6G Parameters recovered from Fluorescence Intensity Decay fitting; Table S6: RB Parameters recovered from Fluorescence Intensity Decay fitting; Table S7: Anisotropy Fitting Results for R6G-Silicate Complex; Table S8: Anisotropy Fitting Results for RhB in Sodium Silicate.
