# Supplementary material for: Nanoparticle Metrology of Silicates Using Time-Resolved Multiplexed Dye Fluorescence Anisotropy, Small Angle X-ray Scattering, and Molecular Dynamics Simulations
Source: Materials (Basel). 2024 Apr 7;17(7):1686. doi: 10.3390/ma17071686 (PMC11012945; doi:10.3390/ma17071686)
Supplement: Supplementary file 1 [file materials-17-01686-s001.zip › materials-2931517-supplementary.pdf]

materials

MDPI

Nanoparticle Metrology of Silicates Using Time-Resolved Multiplexed Dye Fluorescence Anisotropy, Small Angle X-ray Scattering, and Molecular Dynamics Simulations

Daniel Doveiko [1,*], Alan R. G. Martin [2], Vladislav Vyshemirsky [3], Simon Stebbing [4], Karina Kubiak-Ossowska [5], Olaf Rolinski [1], David J. S. Birch [1] and Yu Chen [1,*]

1 Photophysics Group, Department of Physics, University of Strathclyde, Scottish Universities Physics Alliance, Glasgow G4 0NG, UK; djs.birch@strath.ac.uk (D.J.S.B.)
2 EPSRC Future Continuous Manufacturing and Advanced Crystallisation National Facility, University of Strathclyde, 99 George Street, Glasgow G1 1RD, UK; alan.r.martin@strath.ac.uk
3 School of Mathematics and Statistics, University of Glasgow, Glasgow G12 8QQ, UK; vladislav.vyshemirsky@glasgow.ac.uk
4 PQ Silicas UK Limited, Warrington WA5 1AB, UK;
5 ARCHIE-WeSt, Department of Physics, University of Strathclyde, Glasgow G4 0NG, UK;
* Correspondence: daniel.doveiko.2018@uni.strath.ac.uk (D.D.); y.chen@strath.ac.uk (Y.C.)

Supplementary Information

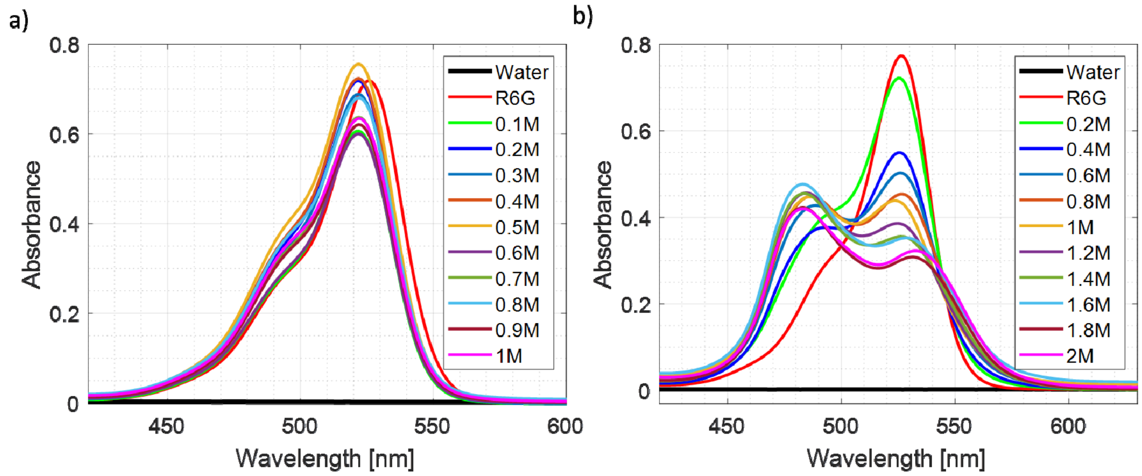

Figure S1. UV-Vis absorption Spectra of (**a**) R6G at different concentrations of NaOH; (**b**) R6G in Sodium silicate containing different concentrations of NaOH.

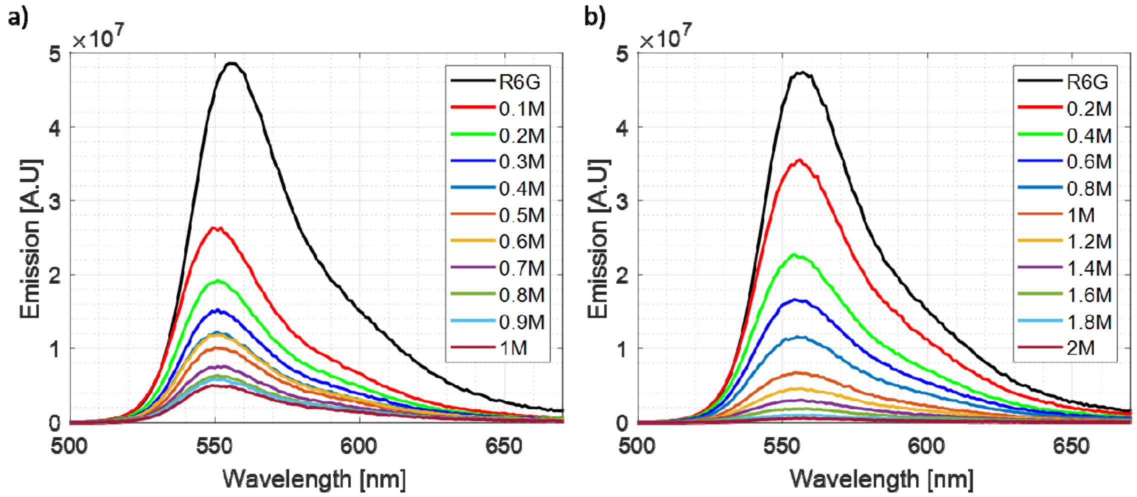

Figure S2. Emission Spectra (λex=494 nm) of (**a**) R6G at different concentrations of NaOH; (**b**) R6G in Sodium silicate containing different concentrations of NaOH.

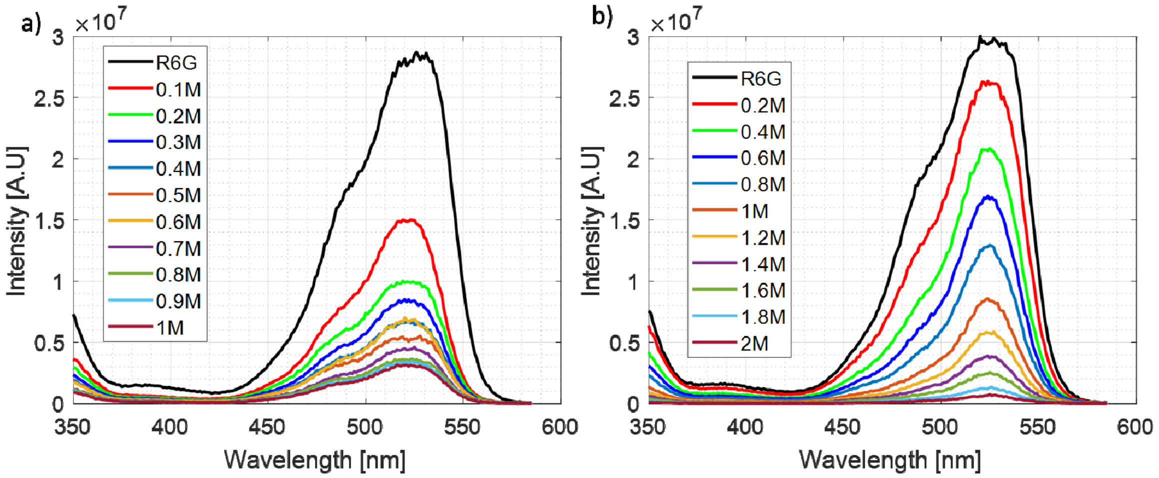

Figure S3. Excitation Spectra (λem=590 nm) of (**a**) R6G at different concentrations of NaOH; (**b**) R6G in Sodium silicate containing different concentrations of NaOH.

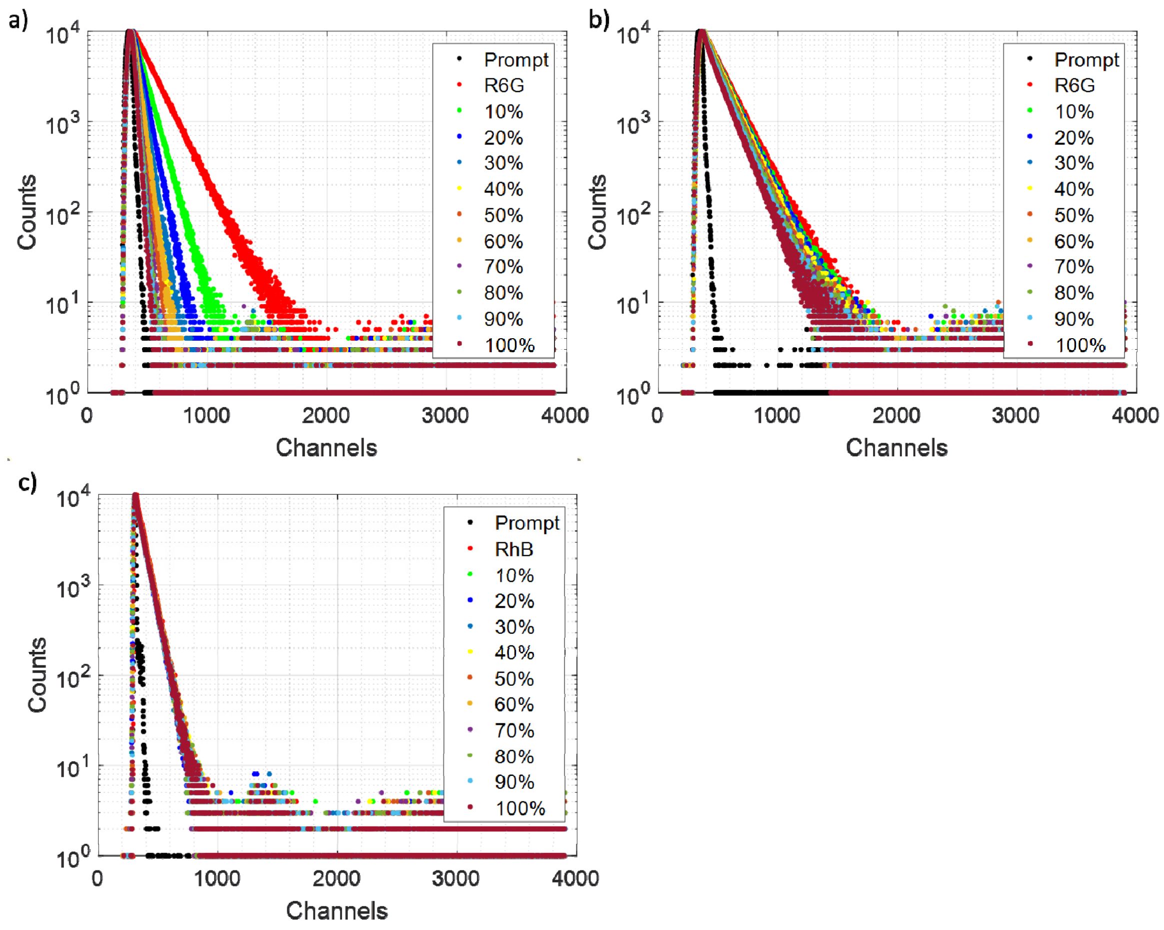

Figure S4. Fluorescence intensity decays of (**a**) R6G in NaOH, (**b**) R6G-silicate complex; (**c**) RB in sodium silicate.

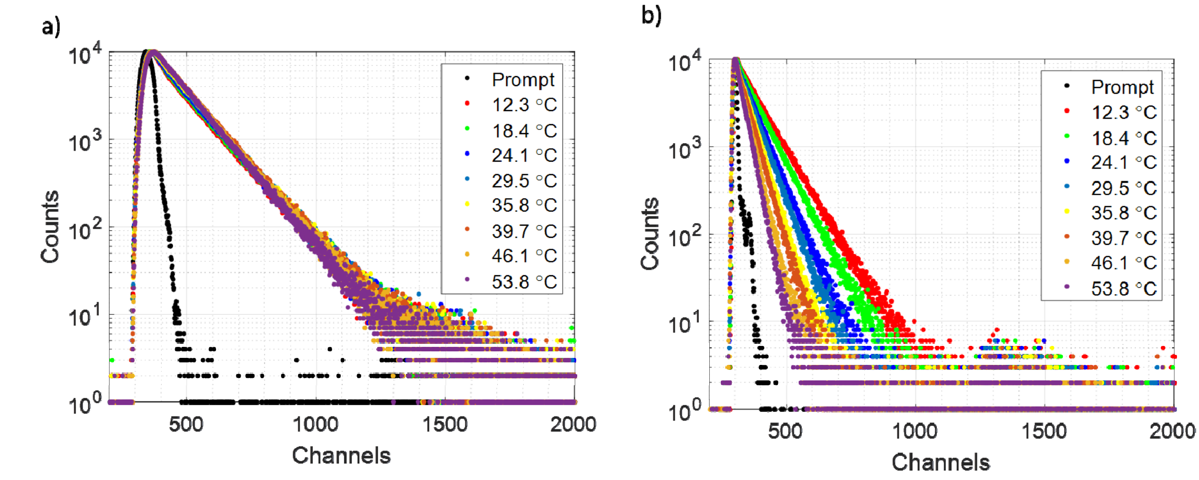

Figure S5. Fluorescence intensity decays at different temperatures (**a**) R6G-Silicate complex; (**b**)RB in Sodium Silicate.

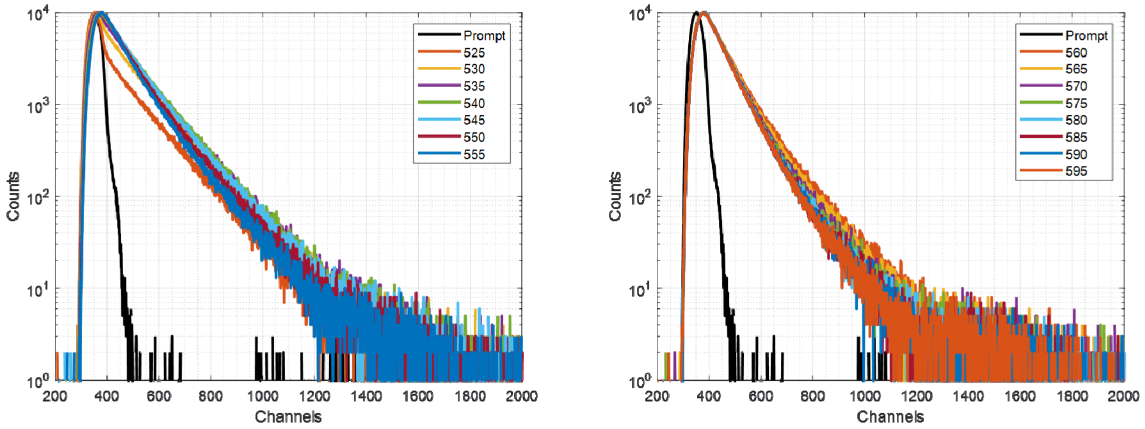

Figure S6. Fluorescence intensity decays for the multiplexed measurements.

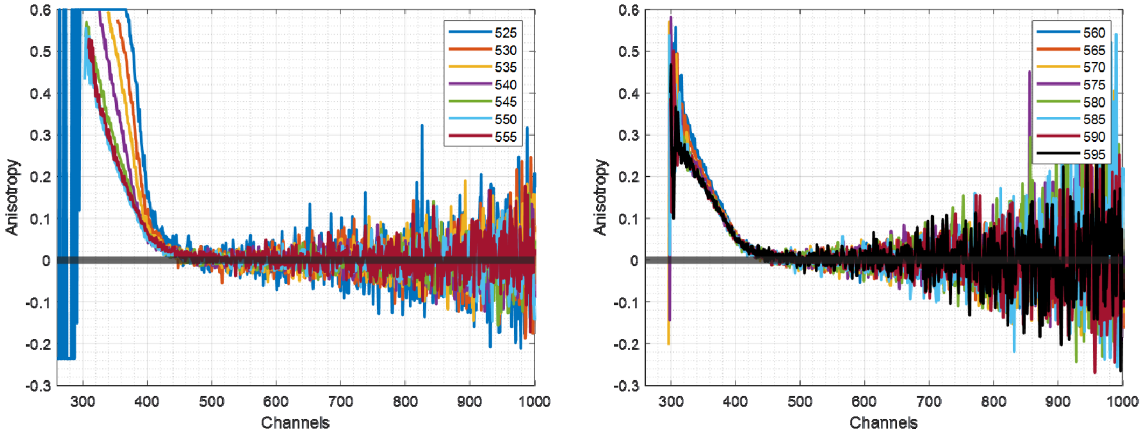

Figure S7. Anisotropy decays for the multiplexed measurements.

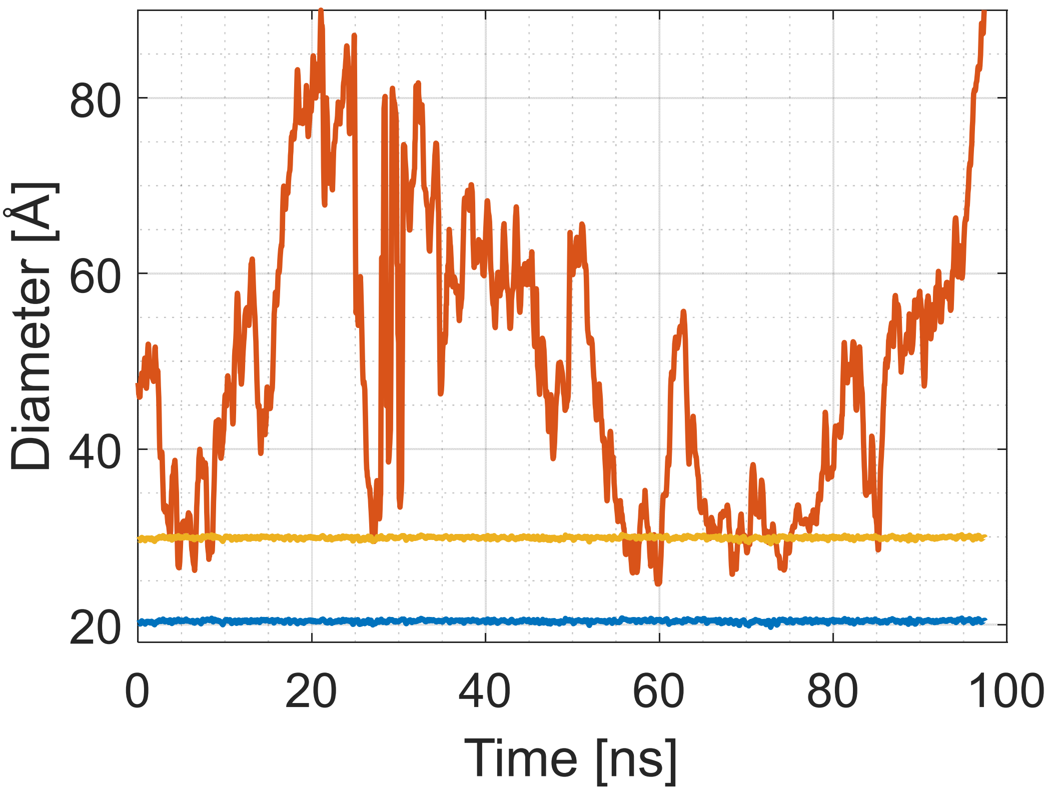

Figure S8. 20 Å diameter SNP measurement results. Colour code follows the one introduced on Figure 9 while the sum of separated sizes is ignored for clarity.

Table S1. R6G Parameters recovered from Fluorescence Intensity Decay fitting.

SiO_2 (%)	[NaOH] M	T1 (ns)	B1 (%)	T2 (ns)	B2 (%)	C (%)	X^2
27.0	2.0	3.42	82.43	0.94	8.01	9.56	1.23
24.3	1.8	3.46	88.30	0.79	5.19	6.50	1.13
21.6	1.6	3.51	93.43	0.58	3.43	3.14	1.18
18.9	1.4	3.55	93.06	0.57	3.59	3.35	1.12
16.2	1.2	3.55	95.23	0.45	3.63	1.15	1.05
13.5	1.0	3.65	94.19	0.59	2.16	3.65	1.09
10.8	0.8	3.73	93.98	0.69	1.14	4.58	1.16
8.1	0.6	3.81	93.39	1.43	1.66	4.95	1.19
5.4	0.4	3.89	93.58	1.78	2.53	3.89	1.18
2.7	0.2	3.98	95.87	0.61	1.40	2.73	1.13
0.0	0.0	4.18	97.20	-	-	2.80	1.07

Table S2. RhB Parameters recovered from Fluorescence Intensity Decay Fitting.

SiO_2 (%)	[NaOH] M	T1 (ns)	B1 (%)	T2 (ns)	B2 (%)	C (%)	X^2
27.0	2.0	1.69	90.62	0.38	5.62	3.76	0.97
24.3	1.8	1.70	90.25	038	5.94	3.81	1.08
21.6	1.6	1.69	92.47	0.24	4.58	2.94	1.04
18.9	1.4	1.69	91.54	0.31	5.36	3.09	0.99
16.2	1.2	1.72	93.32	0.22	3.23	3.45	1.08
13.5	1.0	1.71	94.33	0.30	2.12	3.55	1.04
10.8	0.8	1.73	92.31	0.33	3.78	3.91	1.13
8.1	0.6	1.69	93.84	0.16	2.78	3.38	1.17
5.4	0.4	1.65	93.85	0.22	1.87	4.28	1.16
2.7	0.2	1.68	94.49	0.18	2.03	3.48	0.97
0.0	0.0	1.67	94.15	0.17	2.78	3.06	0.95

Table S3. Anisotropy Fitting Results for R6G-Silicate complex.

SiO_2 (%)	[NaOH] M	Φ (ns)	R0	X^2
27.0	2.0	1.16	0.17	1.10
24.3	1.8	0.84	0.13	1.10
21.6	1.6	0.40	0.31	1.11
18.9	1.4	0.44	0.21	1.05
16.2	1.2	0.27	0.29	1.07
13.5	1.0	0.19	0.33	1.26
10.8	0.8	0.24	0.28	1.08
8.1	0.6	0.17	0.34	1.18
5.4	0.4	0.19	0.22	1.19
2.7	0.2	0.14	0.35	1.12
0.0	0.0	0.16	0.20	1.06

Table S4. Anisotropy Fitting Results for RhB in Sodium Silicates.

SiO_2 (%)	[NaOH] M	Φ (ns)	R0	X^2
27.0	2.0	0.54	0.17	1.00
24.3	1.8	0.46	0.19	1.00
21.6	1.6	0.41	0.20	1.07
18.9	1.4	0.35	0.20	0.96
16.2	1.2	0.32	0.18	1.02
13.5	1.0	0.40	0.15	1.14
10.8	0.8	0.30	0.18	1.22
8.1	0.6	0.25	0.21	1.03
5.4	0.4	0.26	0.15	1.07
2.7	0.2	0.22	0.16	1.19
0.0	0.0	0.22	0.22	1.01

Table S5. R6G Parameters recovered from Fluorescence Intensity Decay fitting.

T (˚C)	T1 (ns)	B1 (%)	T2 (ns)	B2 (%)	C (%)	X^2
12	3.47	75.88	1.01	10.73	10.72	1.19
18	3.45	81.34	0.89	9.40	9.27	1.29
24	3.42	84.90	0.82	6.30	8.80	1.20
29	3.41	86.69	0.96	5.31	8.00	1.21
35	3.35	89.23	0.95	3.01	7.75	1.23
39	3.33	90.74	1.16	3.06	6.21	1.18
46	3.22	92.52	0.88	2.09	5.38	1.15
53	3.11	94.46	-	-	5.54	1.18

Table S6. RB Parameters recovered from Fluorescence Intensity Decay fitting.

T (˚C)	T1 (ns)	B1 (%)	T2 (ns)	B2 (%)	C (%)	X^2
12	2.25	87.51	0.59	8.73	3.76	1.07
18	1.93	88.01	0.48	8.04	3.94	0.98
24	1.56	88.30	0.35	6.73	4.97	1.19
29	1.39	87.98	0.33	5.88	6.14	1.01
35	1.15	87.13	0.27	6.85	6.02	1.24
39	1.06	82.52	0.43	9.18	8.30	1.21
46	0.90	68.53	0.47	19.91	11.57	1.09
53	1.13	7.51	0.65	79.36	13.14	1.25

Table S7. Anisotropy Fitting Results for R6G-Silicate Complex.

T (˚C)	Φ (ns)	R0	X^2
12	1.17	0.17	1.11
18	0.76	0.13	1.06
24	0.82	0.10	1.09
29	0.51	0.15	1.05
35	0.44	0.17	1.14
39	0.32	0.22	1.05
46	0.18	0.34	1.10
53	0.22	0.25	1.05

Table S8. Anisotropy Fitting Results for RhB in Sodium Silicate.

T (˚C)	Φ (ns)	R0	X^2
12	0.72	0.17	1.15
18	0.53	0.17	1.12
24	0.54	0.12	1.10
29	0.37	0.13	1.12
35	0.23	0.12	1.16
39	0.29	0.11	1.15
46	0.19	0.10	1.18
53	0.14	0.09	1.16
